# A Comparison of Home Health Care Between Finland and South Korea in Long-Term Care Services

**DOI:** 10.1093/geroni/igaa057.269

**Published:** 2020-12-16

**Authors:** Jihyun Park, Suhyun Kim

**Affiliations:** 1 Yonsei University, Seoul, Republic of Korea; 2 Kyungpook National University, Daegu, Republic of Korea

## Abstract

Population aging is one of the significant global issues. Long-term care is emphasized as “aging in place,” and it is known that home and community-based service is a cost-effective way to achieve this. Over ten years have passed since the introduction of long-term care insurance in Korea, and it is necessary to improve home health care in long-term care. The aim of this study was to identify the measures that must be undertaken for enhancing home health care in Korea by comparing it to the home health care in Finland. The data were collected via a literature review, expert interview, and field survey in Kuopio, Eastern Finland, from March 16 to 23, 2018. Based on the comparison between Korean and Finnish home health care, some issues related to home health care in Korea that need to be resolved were identified: the complex process involved in availing home health care, low utilization rate, higher cost than home health aide services in long-term care, and undifferentiated roles in home health care between registered nurses and nurse assistants. Several strategies could be utilized to enhance home health care in Korea, such as a simplified procedure to use home health care, clarification of roles between registered nurses and nurse assistants in home care, supervision of the integration of home care services by registered nurses, and an expansion of home health care into comprehensive assessment and nursing activities for chronic illness care and health promotion.

